# Trade-Offs between Economic and Environmental Impacts of Introducing Legumes into Cropping Systems

**DOI:** 10.3389/fpls.2016.00669

**Published:** 2016-05-23

**Authors:** Moritz Reckling, Göran Bergkvist, Christine A. Watson, Frederick L. Stoddard, Peter M. Zander, Robin L. Walker, Aurelio Pristeri, Ion Toncea, Johann Bachinger

**Affiliations:** ^1^Institute of Land Use Systems, Leibniz Centre for Agricultural Landscape ResearchMüncheberg, Germany; ^2^Department of Crop Production Ecology, Swedish University of Agricultural SciencesUppsala, Sweden; ^3^Crop and Soil Systems, Scotland's Rural CollegeAberdeen, UK; ^4^Department of Agricultural Sciences, University of HelsinkiHelsinki, Finland; ^5^Institute of Socio-Economics, Leibniz Centre for Agricultural Landscape ResearchMüncheberg, Germany; ^6^Department of Agricultural Science, Mediterranean University of Reggio CalabriaReggio Calabria, Italy; ^7^National Agricultural Research and Development InstituteFundulea, Romania

**Keywords:** crop rotation, framework, land use and impacts, multi-criteria assessment, protein crops, resource-efficiency, rotation generator

## Abstract

Europe's agriculture is highly specialized, dependent on external inputs and responsible for negative environmental impacts. Legume crops are grown on less than 2% of the arable land and more than 70% of the demand for protein feed supplement is imported from overseas. The integration of legumes into cropping systems has the potential to contribute to the transition to a more resource-efficient agriculture and reduce the current protein deficit. Legume crops influence the production of other crops in the rotation making it difficult to evaluate the overall agronomic effects of legumes in cropping systems. A novel assessment framework was developed and applied in five case study regions across Europe with the objective of evaluating trade-offs between economic and environmental effects of integrating legumes into cropping systems. Legumes resulted in positive and negative impacts when integrated into various cropping systems across the case studies. On average, cropping systems with legumes reduced nitrous oxide emissions by 18 and 33% and N fertilizer use by 24 and 38% in arable and forage systems, respectively, compared to systems without legumes. Nitrate leaching was similar with and without legumes in arable systems and reduced by 22% in forage systems. However, grain legumes reduced gross margins in 3 of 5 regions. Forage legumes increased gross margins in 3 of 3 regions. Among the cropping systems with legumes, systems could be identified that had both relatively high economic returns and positive environmental impacts. Thus, increasing the cultivation of legumes could lead to economic competitive cropping systems and positive environmental impacts, but achieving this aim requires the development of novel management strategies informed by the involvement of advisors and farmers.

## Introduction

Crop production in Europe is highly specialized and currently relies on a very small number of crop species, raising questions about the sustainability of farming (Tilman et al., 2002). Furthermore, less than 30% of the demand for protein feed supplement is produced within Europe (Bouxin, 2014). The reintegration of legumes into European agriculture could reduce the current protein deficit and contribute to the transition to more sustainable agricultural systems (Voisin et al., 2013).

The use of legumes also affects the performance of cropping systems and their environmental impacts, including (i) nitrogen supply via biological nitrogen fixation (BNF), reducing the demand for external nitrogen fertilizers, (ii) positive pre-crop benefits through a combination of residual nitrogen and break-crop effects (Angus et al., 2015; Preissel et al., 2015), (iii) reduced fossil energy consumption in crop production (Jensen et al., 2011), and (iv) increased crop diversification and biodiversity (Köpke and Nemecek, 2010).

Despite these benefits, there are good reasons why European farmers grew grain legumes on only 1.6% of the arable land in 2014 (FAOstat, 2015). These include specialization in a few crops (Zander et al., 2016), low and unstable yields of grain legumes (Von Richthofen et al., 2006; Cernay et al., 2015; Reckling et al., 2015), low and unpredictable policy support (Bues et al., 2013), and lack of awareness of, and inability to evaluate the benefits of including legumes in the cropping system (Preissel et al., 2015). As a consequence, the area under grain legumes declined from 4.7% of arable land in 1961 to currently below 2% (FAOstat, 2015). Organic farms used 6.8% of their arable land for production of grain legumes in 2014 (Eurostat, 2015). Forage legume areas also declined because of a transformation of the ruminant feeding system toward maize silage and soybean oil cakes, and geographical separation between cereal and cattle-rearing areas (Voisin et al., 2013).

By introducing legumes into the cropping system, farmers also introduce more complexity into their planning. To maximize the benefits of legumes while minimizing potential threats, a number of issues need to be taken into account. The preceding crop in a rotation should have a high ability to take up available nitrogen in order to reduce residual soil N levels and allow BNF of the legumes to be optimized. Legume crops need to be kept several years apart in the rotation because of their susceptibility to soil-borne diseases. The following crop should take up the residual N of the legume quickly, in order to minimize opportunities for nitrate leaching and nitrous oxide emission. Legume crops, their management and their effects on other crops in the rotation are specific to different climatic and edaphic conditions (Döring, 2015), e.g., narrow-leafed lupin (*Lupinus angustifolius* L.) is considered suitable for sandy soils with low soil pH, whereas faba bean (*Vicia faba* L.) is typically grown on clay soils with sufficient rainfall, soybean (*Glycine max* (L.) Merr.) requires appropriate daylength to flower and sufficient growing degree-days to mature, and pea (*Pisum sativum* L.) is suitable for many different soil types. Therefore, the design of cropping systems needs to be site-specific and based on regional expert knowledge.

Although the positive effects of legumes on subsequent crops, including yield benefits and reductions in nitrogen fertilizer demand, soil tillage and biocide applications are well known (Angus et al., 2015; Preissel et al., 2015) from field experiments, these effects are difficult to formalize and are rarely considered in modeling (Bergez et al., 2010). Life cycle assessments comparing rotations with and without legumes have been carried out (e.g., Nemecek et al., 2008; Köpke and Nemecek, 2010) but these work with a limited set of rotations and do not include the detailed levels of field management required to evaluate all rotational effects.

In order to focus research and policy intervention, a method is needed to assess the economic and environmental effects of changes to cropping system design, such as the integration of legumes. To identify novel practices that achieve high economic and environmental benefits the method should provide a range of crop combinations and evaluate management options beyond current farming practices.

We earlier (Reckling et al., 2016) formulated an assessment framework that allows a systematic analysis of a large range of cropping options. It operates on the cropping system scale and takes rotational effects of crops such as legumes into account.

The objective of this study is to assess the economic and environmental effects of integrating legumes into cropping systems, in order to identify potentials and limitations of increasing legume cultivation in Europe. For the analysis we apply the cropping system framework by Reckling et al. (2016) in case studies in Germany, Italy, the United Kingdom, Romania, and Sweden. We hypothesize that the integration of legumes increases positive environmental impacts and at the same time reduces economic returns, thus leading to greater trade-offs between economic and environmental benefits.

## Methods

The static and rule-based framework developed by Reckling et al. (2016) was used to assess cropping systems in five case studies following three steps: (i) *generate crop rotations* (using a rule-based rotation generator), (ii) *calculate the impact of crop production activities* (using environmental and economic indicators), and (iii) *assess and compare cropping systems* with and without legumes. Following Bergez et al. (2010), we used a multi-criteria analysis for the identification of trade-offs.

### Case studies

**Figure 1:**
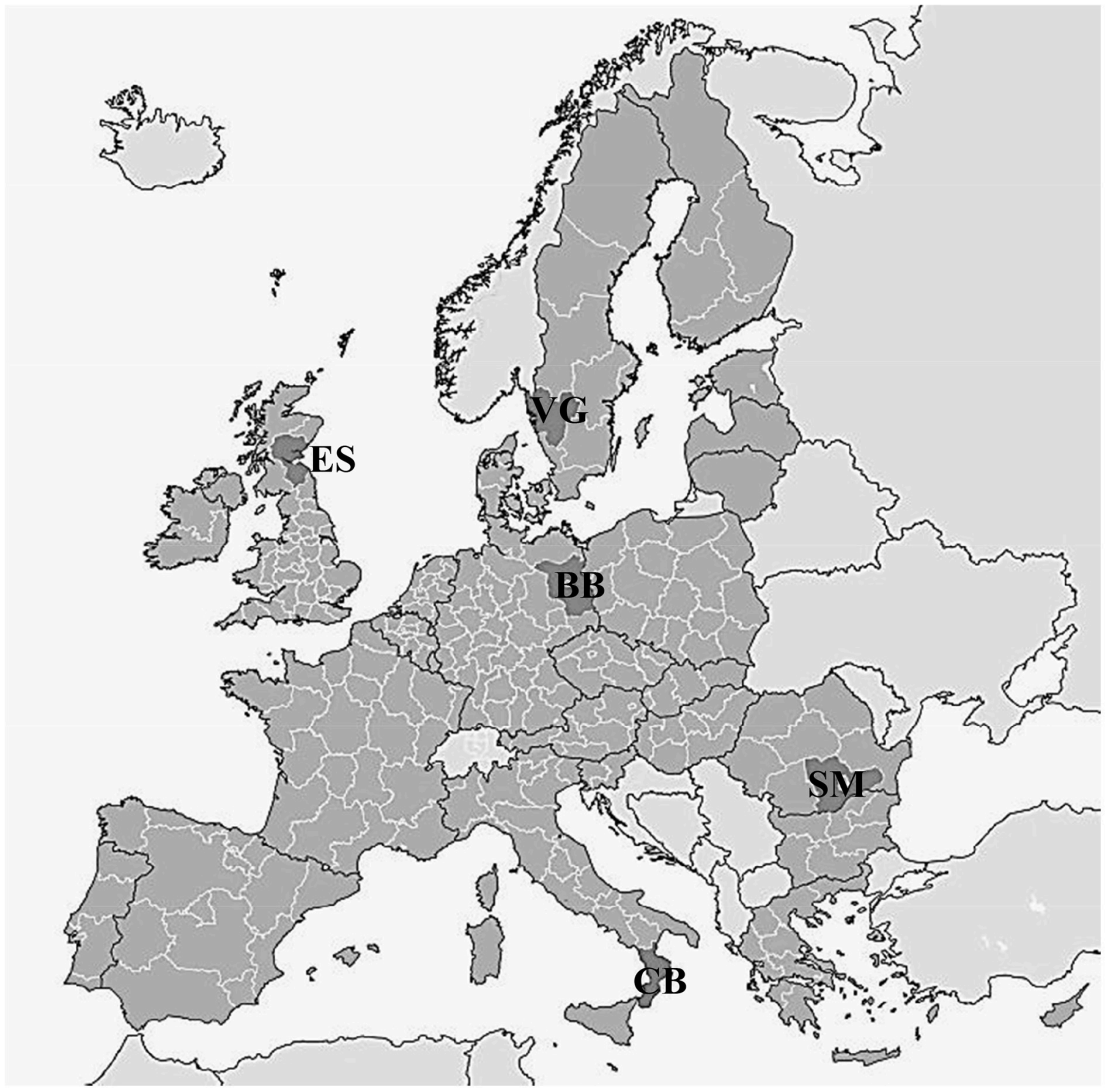
**Case study regions across Europe, Brandenburg (BB) in north-eastern Germany, Calabria (CB) in southern Italy, eastern Scotland (ES) in the United Kingdom, Sud-Muntenia (SM) in Romania, and Västra Götaland (VG) in western Sweden (adapted from Eurostat 2010)**.

Five contrasting regions were selected across Europe (Figure 1): Brandenburg (BB) in north-eastern Germany, Calabria (CB) in southern Italy, eastern Scotland (ES) in the United Kingdom, Sud-Muntenia (SM) in Romania, and Västra Götaland (VG) in western Sweden. The regions are characterized by contrasting climatic conditions and cropping systems, and were selected to represent a broad range of bio-physical and socio-economic conditions and possible roles of legume production. In all regions, legumes occupy less than 2.5% of the arable land. Within case studies, land capability types were defined by regional classifications and differences in soil texture (Table 1). These determine the choice of the crops, their management and performance.

**Table 1:** **Cropping characteristics of selected regions and land capability types**.

**Region**	**Site**	**% sand**	**% clay**	**% of agric. area**	**Non-legume crops**	**Legume crops**
**ARABLE CROPPING SYSTEMS**						
BB	Type 2	50	25	22	WW, WR, SB, WB, WT, WOR, SM, SO	FP, FB
CB	Type 3	47	23	36	WB, DW, SO, WOR, WW, WT	FP, FB
ES	Type 1/2	78	14	21	SB, WB, SO, WO, SOR, WOR, SW, WW	FP, FB
SM	Type 1	65	18	42	WW, WOR, GM, SF, WB	FP, CB, SY
VG	Type 1	17	33	50	SB, SO, SW, WW, SOR, WOR, WR, WT	FP, FB
**FORAGE CROPPING SYSTEMS**						
BB	Type 3	70	20	37	WW, WR, WOR, SM, SO, SB	FP, LU, GC, AF
ES	Type 3	68	17	10	SB, WB, SOR, WOR, SW, WW, SO, WO, GR	GC
VG	Type 1	17	33	50	SM, SB, SO, SW, WW, SOR, WOR, WR, WT, GR	FP, FB, FP-SO, GC

BB, Brandenburg; CB, Calabria; ES, Eastern Scotland; SM, Sud-Muntenia; and VG, Västra Götaland. Types represent different land capabilities. AF, Alfalfa; CB, common bean; DW, durum wheat; FB, faba bean; FP, field pea; GC, grass-clover; GM, grain maize; GR, grass; LU, lupin; SB, spring barley; SF, sunflower; SM, silage maize; SO, spring oat; SOR, spring oilseed rape; SW, spring wheat; SY, soybean; WB, winter barley; WO, winter oat; WOR, winter oilseed rape; WR, winter rye; WT, winter triticale; WW, winter wheat.

In Brandenburg, soil types are classified according to LELF (2010). Arable framing concentrates on Type 1–2 with higher yield potential and forage oriented farming on Type 3–4 with a lower yield potential. Soil Type 2 was selected for arable and Type 3 for forage systems. In Calabria, the assessment focused on arable rainfed systems in the lowlands, where pea and faba bean are the main annual legume crops. In Eastern Scotland, mixed cropping is common across soil Types 1–3, and forage production on Types 3 and 4 (classified according to Bibby et al., 1982). For the assessment, Type 1 and 2 were selected for arable and Type 3 for forage systems. Cereals occupy 23% and grassland 73% of the agricultural land, while faba bean and pea together account for only 0.3% (Scottish-Agricultural-Census, 2010). Arable farmers in the lowlands grow large shares of cereals and relatively few break crops, mainly winter and spring oilseed rape, potato, and swede. In the hilly areas, farms are dominated by grassland with low shares of clover. In Sud-Muntenia, Chernozem soils with high humus contents were selected, as they underlie the major cropping areas with medium- and large-scale farms. The study considered only arable farms, because information on forage grass production was limited. Legumes already grown include pea and soybean for feed and common bean (*Phaseolus vulgaris* L.) for the food market. In Västra Götaland, soils with high clay contents were selected for this study, because they represent the major cropping areas with both arable and mixed farms. Arable farmers use 80–100% of their land for cereals, and legumes and other break crops play minor roles (1.7 and 1.5% of the arable land). In forage-oriented farms, perennial grasses with a relatively low share of legumes (< 20%) are common.

Across the case studies, pea, faba bean and clover (*Trifolium* L.) (sole cropped or in mixture with grasses) were the most widely tested legumes. Wheat, barley and oilseed rape were the most common non-legume crops (Table 1).

### Generate crop rotations

Crop rotations were generated with the previously described static and rule-based rotation generator (Reckling et al., 2016) that produces fixed and cyclical “agronomically sound rotations” for arable fields, following a fixed set of site-specific agronomic rules. In each region, agronomists defined suitable crops and then the restriction values for crop sequence, including suitability of crop-crop combinations, the minimum cultivation break and frequency restrictions (% of a crop and crop type in a rotation) to allow the site-specific generation of rotations. In addition, a rotation of *current farming* (“business as usual”) was based on the most common crops grown.

The rotation generator combined crops to produce all possible 3- to 6-year sequences following the criteria (i) crop sequence suitability (e.g., pea-rye), (ii) maximum frequency of a crop in the rotation (e.g., 20% for pea), (iii) minimum break between the same crop (4 years for pea), and (iv) maximum frequency of crops of the same crop type (e.g., 25% grain legumes). Duplicates and multiples were removed and rotations were filtered by soil type. Agronomists checked the plausibility of the results in comparison to the existing sequences.

### Calculate impact indicators

The crop production activities (CPA) covered the crop, preceding crop and site-specific crop management. CPA were designed by collecting management data in a structured survey among 2–4 experienced agronomists in each case study in 2013. The data included inputs (seed, N-P-K fertilizer and pesticides), outputs (grain yield, forage yield, and straw yield) and management characteristics (fertilizer intensity, machinery use, and harvesting method) for each crop. In order to consider rotational effects, pre-crop types were defined according to their ability to influence the yield of the following crop through N from residue decomposition. Besides the effect on yield, differences in fertilization and agro-chemical applications were considered for each pre-crop type. The following types were separated, in the order of increasing N residue levels and positive effect on yield of subsequent crops: (i) spring cereals, winter cereals and maize; (ii) grain legumes, rapeseed and grass (< 10% legumes); (iii) forage legumes in pure stands and in mixtures with grass (>30% legumes).

We selected five indicators to assess the economic and environmental performance of cropping systems. Nitrogen is not only important in crop production, it also contributes to environmental pollution of the atmosphere and water bodies, so nitrous oxide emission and nitrate leaching were selected as indicators. Nitrogen fertilizer use and nitrogen fertilizer efficiency were selected as indicators of resource use efficiency. Gross margins provided the standard indicator for economic performance of crops. All impacts were calculated on a per-hectare and per-year basis.

#### Calculation of nitrate leaching

Nitrate leaching is calculated as nitrate-N based on the soil type, preceding crop and crop management as a function of the soil leaching probability and the nitrogen surplus. Adapted from Gäth and Wohlrab (1992) and Bachinger and Zander (2007), nitrate leaching is calculated as:

$$N_{leaching}=N_{surplus}*L_{P}$$

where *L*_*P*_ is the leaching probability during the winter (mean winter precipitation divided by the water holding capacity at rooting depth and a crop-specific leaching coefficient) and the N_*surplus*_ is calculated as:

$$N_{surplus}=\left(N_{manureP}+N_{fertilizer}+N_{mineralization}-N_{dfs}\right)$$

where *N*_*manureP*_ is the plant-available N content in solid and liquid manure and *N*_*mineralization*_is calculated as a function of the total organic N content (typical contents per soil type) and a region-specific mean annual mineralization rate modified by the pre-crop specific N supply level. *N*_*dfs*_ is the nitrogen derived from soil and is calculated as:

$$N_{dfs}=N_{uptake}-N_{fixation}$$

Where *N*_*uptake*_ is the nitrogen accumulated by the crop and *N*_*fixation*_ is BNF of grain and forage legumes calculated as a function of the yield, the N content, the crop-specific ratio of above and below ground plant N, the percentage of N derived from the atmosphere (%Ndfa) depending on soil mineral N content using minimum and maximum %Ndfa values (Peoples et al., 2009), the percentage of legumes in crop mixtures (grass-clover and cereal-legume intercropping), and the ratio of fixed N transferred to grass in grass-clover mixtures. The soil mineral N content is estimated taking into account possible N mineralization from preceding crop residues in spring, along with N inputs from plant-available N in manure and mineral N fertilizer (Reckling et al., 2016). Atmospheric nitrogen deposition and non-symbiotic nitrogen fixation were not considered because reliable data was not available.

#### Calculation of nitrogen fertilizer efficiency and use

Nitrogen fertilizer efficiency was calculated as the ratio of the N output in harvested grain or biomass to the N input from mineral and organic fertilizer and N in seed. N fertilizer use was calculated as the average N applied from organic and mineral N fertilizer.

#### Calculation of nitrous oxide emissions

Soil-based nitrous oxide emission from crop cultivation was calculated with the IPCC 2006 Tier 1 methodology, including direct and indirect emission from applied fertilizer, manure and crop residues using standard factors and parameters, and assuming no emission from nitrogen fixation (IPCC, 2006).

#### Calculation of gross margins

Gross margins are calculated by subtracting variable costs (inputs, variable costs of machinery and services) from the revenues (yield multiplied by the product price). Labor costs, subsidies, interest and insurance were not taken into account. Data for the economic analysis were obtained from regional statistics (KTBL, 2010; LELF, 2010; Scottish-Agricultural-Census, 2010; SJV, 2011; ISTAT, 2013; MADR, 2013; SAC, 2013; Eurostat, 2015).

### Analysis of cropping systems

Cropping systems were generated for each land capability and evaluated using the impact indicators. The evaluated systems were grouped as either *arable*, including only grain crops, or *forage*, including at least one forage crop, and the groups were further divided into those *with legumes* or *without legumes*. The dataset was analyzed by plotting environmental impacts against economic impacts of all single cropping systems and by calculating the mean impact and difference between arable and forage systems with and without legumes.

Trade-offs were analyzed between economic and environmental impacts using multi-criteria analysis based on five indicators: (i) Gross margin, (ii) nitrate leaching, (iii) nitrous oxide emission, (iv) N fertilizer use, and (v) N fertilizer efficiency. To allow relative comparisons, impacts were normalized for each land capability and arable and forage systems separately by dividing the impact of each single cropping system by the overall mean. Trade-offs were analyzed for selected cropping systems:

1. *current farming* without legumes
2. *economic-environmental optimized* systems without legumes
3. *economic-environmental optimized* systems with legumes

*Current farming* without legumes represents the most common cropping system per land capability based on the crop proportions and expert knowledge. *Economic-environmental optimized* cropping systems with and without legumes are selected from the large range of generated systems according to the criteria:

- Gross margin is equal to or 50€ lower than the system with the highest gross margin
- Nitrate leaching is equal to or lower than the system with the highest gross margin
- Nitrous oxide emission is equal to or lower than system with the highest gross margin

The selected cropping systems were compared in radar charts to identify trade-offs and to evaluate the performance from a systems perspective.

### Model evaluation

Generated rotations were compared by agronomists and advisors with current and plausible cropping patterns. Algorithms for nitrate leaching were already validated by Bachinger and Zander (2007) using HERMES, a dynamic model that simulates water and soil nitrogen dynamics (Kersebaum, 1995). Model outputs for each land capability type were compared against published data and by experts in nitrate leaching. The IPCC method to estimate nitrous oxide emissions is acknowledged to be simple and static, so is open to criticism (Philibert et al., 2012). Nevertheless, it is widely used in GHG inventories and emission models (Lokupitiya and Paustian, 2006; Berdanier and Conant, 2012) because it can be relatively easily applied with only a few input parameters. The model outputs were checked by a specialist in nitrous oxide emission measurements and models (B. Rees, pers. communication). Calculations of impacts at the cropping systems scale were evaluated by a panel of 12 agronomists who discussed the generated systems and compared the results with current management practices in their regions.

## Results

Integration of legumes affected the economic and environmental performance of cropping systems. The environmental performance was evaluated in terms of reduced leaching and nitrous oxide loss and lower fertilizer use, and economic performance was evaluated in terms of higher gross margin. On average across all regions, arable cropping systems with legumes had 18% less nitrous oxide emission, 24% less N fertilizer use, no increase in nitrate leaching and no decrease in gross margin (Table 2). In forage cropping systems, average effects were stronger, namely 33% less nitrous oxide emission, 38% less N fertilizer use, 22% less nitrate leaching and 21% higher gross margin (Table 2). Effects varied between the case studies due to the different crop management, bio-physical and socio-economic conditions, and between arable and forage systems.

**Table 2:** **Average economic and environmental impacts for cropping systems with and without legumes**.

**Region**	**Site**	**Legume**	**Generated systems [no.]**	**Gross margin [€/ha]**	**NO_3_-N leaching [kg/ha]**	**N fertilizer use [kg/ha]**	**N_2_O emissions [kg/ha]**
**ARABLE CROPPING SYSTEMS**							
BB	Type 2	+legume	249	14	21	88	3.0
		-legume	68	51	23	114	3.6
CB	Type 1	+legume	328	195	24	32	1.3
		-legume	12	263	23	53	1.9
ES	Type 1/2	+legume	1237	637	23	107	4.1
		-legume	227	600	30	132	4.6
SM	Type 1	+legume	220	476	13	86	3.0
		-legume	20	369	13	108	3.6
VG	Type 1	+legume	10,127	420	30	100	3.5
		-legume	1756	452	31	121	4.0
**FORAGE CROPPING SYSTEMS**							
BB	Type 3	+legume	102	130	18	53	2.2
		−legume	89	80	37	126	4.7
ES	Type 3	+legume	18	733	24	220	7.4
		-legume	23	715	30	311	9.7
VG	Type 1	+legume	146	482	15	146	4.7
		-legume	108	483	14	201	6.1

BB, Brandenburg; CB, Calabria; ES, Eastern Scotland; SM, Sud-Muntenia; and VG, Västra Götaland. Types represent different land capabilities.

### Effects of integrating legumes into arable cropping systems

**Figure 2:**
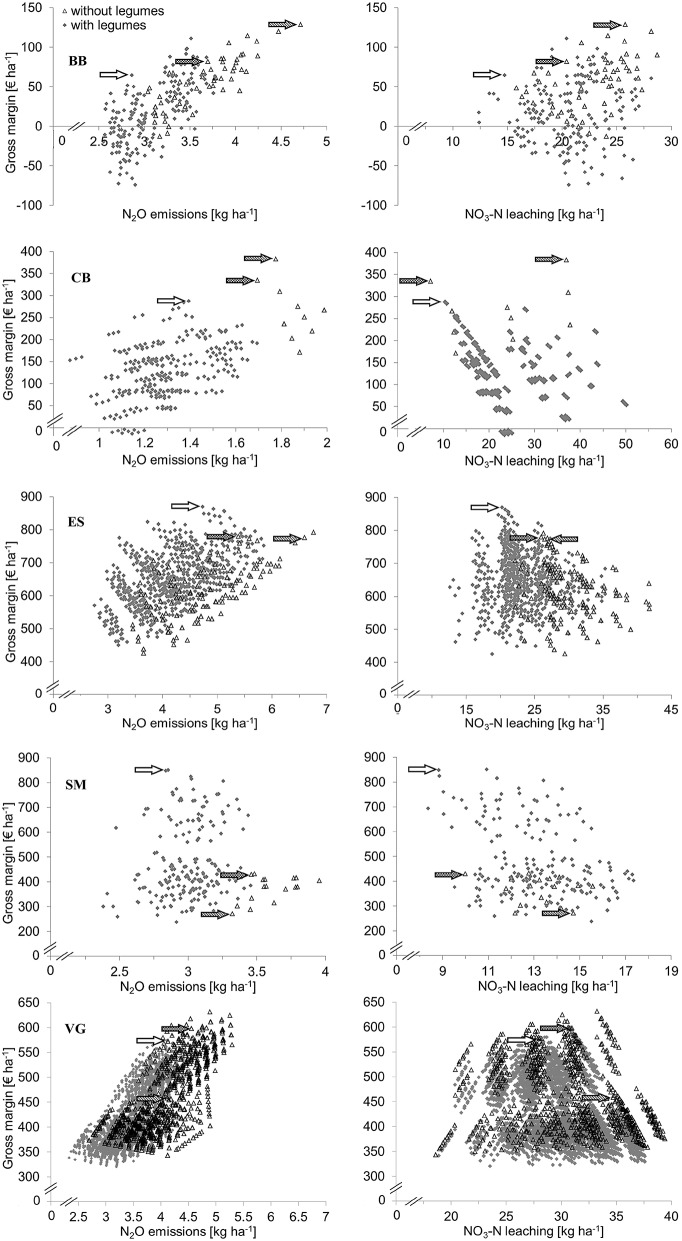
**Gross margin plotted against N_2_O emissions and NO_3_-N leaching for arable cropping systems with and without legumes in Brandenburg (BB), Calabria (CB), Eastern Scotland (ES), Sud-Muntenia (SM), and Västra Götaland (VG)**. Blank and dotted arrows indicate economic-environmental optimized systems with and without legumes and dashed arrows indicate current farming.

The inclusion of grain legumes, such as pea and faba bean, in arable cropping systems reduced N fertilizer use by 17–40% and nitrous oxide emission by 12–30% (Table 2). The smallest effects were found in VG for N fertilizer use and ES for nitrous oxide emission and the greatest in CB for N fertilizer use and nitrous oxide emission. Gross margins were 6 and 29% higher in ES and SM, and 7, 26, and 73% lower in VG, CB and BB, respectively (Table 2). Nitrate leaching was similar or slightly reduced in arable systems. Cropping systems with high gross margin tended to have higher nitrous oxide emissions than cropping systems with low gross margin. No relationship was observed between gross margin and nitrate leaching (Figure 2).

The *current farming* scenario without legumes achieved high economic returns in BB, CB and ES and resulted in relatively high nitrate leaching and nitrous oxide emission in all regions (Figure 2). In VG and SM the *current farming* system achieved a low economic and environmental performance although it is practiced by a large share of farmers according to crop share statistics. The inclusion of oilseed rape improved gross margins in BB and VG and environmental benefits in cropping systems with and without legumes. The *economic-environmental optimized* system without legumes performed economically better than *current farming* in ES, SM and VG and environmentally better in all regions (Table 3).

**Table 3:** **Economic and environmental impacts for current and optimized cropping systems with and without legumes**.

**Region**	**Site**	**System**	**Legume**	**Crop 1**	**Crop 2**	**Crop 3**	**Crop 4**	**Crop 5**	**Crop 6**	**Gross margin [€/ha]**	**NO_3_-N leaching [kg/ha]**	**N fertilizer use [kg/ha]**	**N_2_O emissions [kg/ha]**
**ARABLE CROPPING SYSTEMS**													
BB	Type 2	Current	−legume	WW	WB	OR				128	26	139	4.7
		Econ.−env. optimized	−legume	WR	WR	WR	SB	OR		82	20	125	3.7
		Econ.−env. optimized	+legume	WW	WR	WR	WR	**FP**		64	15	94	2.9
CB	Type 1	Current	−legume	WB	WO	WB	WO			383	37	49	1.8
		Econ.−env. optimized	−legume	WB	OR	WB	OR			335	7	46	1.7
		Econ.−env. optimized	+legume	WB	OR	WB	**FB**	OR		287	10	34	1.4
ES	Type 1/2	Current	−legume	WB	OR	WW	WW	WB		776	27	192	6.6
		Econ.−env. optimized	−legume	WB	OR	WB	WO	SB		780	26	156	5.4
		Econ.−env. optimized	+legume	WB	OR	WB	WO	**FP**		869	20	130	4.7
SM	Type 1	Current	−legume	GM	WW	SF				272	15	102	3.3
		Econ.−env. optimized	−legume	GM	WB	OR				430	10	103	3.5
		Econ.−env. optimized	+legume	GM	WB	OR	**CB**			848	9	80	2.8
VG	Type 1	Current	−legume	WW	WW	SO				459	35	124	4.1
		Econ.−env. Optimized	−legume	WW	SB	OR	WR	SB		598	31	143	4.6
		Econ.−env. optimized	+legume	WW	SB	OR	WR	**FB**		573	28	126	4.1
**FORAGE CROPPING SYSTEMS**													
BB	Type 3	Current	−legume	SM	SM	SM	WR			262	40	166	6.9
		Econ.−env. optimized	−legume	SM	WR	SM	SO	WR		131	34	131	4.8
		Econ.−env. optimized	+legume	**GC**	**GC**	WR	WR	WR	SB	184	10	60	2.0
ES	Type 3	Current	−legume	GR	GR	GR	SB	SO		664	29	293	9.1
		Econ.−env. optimized	−legume	GR	GR	GR	SB			767	18	340	10.5
		Econ.−env. optimized	+legume	**GC**	**GC**	**GC**	SB			795	12	235	8.0
VG	Type 1	Current	−legume	GR	GR	GR	WW	SO		535	12	222	6.6
		Econ.−env. optimized	−legume	GR	GR	GR	OR	WT	SO	551	12	212	6.3
		Econ.−env. optimized	+legume	**GC**	**GC**	**GC**	OR	WR	SO	567	13	163	5.0

BB, Brandenburg; CB, Calabria; ES, Eastern Scotland; SM, Sud-Muntenia; and VG, Västra Götaland. Types represent different land capabilities. CB, Common bean; FB, faba bean; FP, field pea; GC, grass-clover; GM, grain maize; GR, grassland; OR, winter oilseed rape; SB, spring barley; SF, sunflower; SM, silage maize; SO, spring oat; WB, winter barley; WO, winter oat; WR, winter rye; WW, winter wheat; legumes are highlighted in bold.

The *optimized* system with legumes achieved 12 and 97% higher gross margins in ES and SM than the *optimized* system without legumes, because of the high yield and high market value (for food use) of pea and common bean in the two regions (Table 3). Besides, adding a legume as another break crop to the cropping system led to an increased yield of the subsequent crop and hence to a higher gross margin. In VG, CB, and BB the gross margin was 4–21% lower in the *optimized* system with legumes than in the *optimized* system without legumes, mainly due to low yields and low prices for pea and faba bean (Table 3). The *optimized* system with legumes had 12–25% lower N fertilizer use, 9–28% lower nitrate leaching (except in CB) and 9–23% lower nitrous-oxide emissions than that without legumes (Table 3). The major contributory factors to the improved environmental effects were that no N fertilizer was applied to the legume crop, the N fertilizer dose to the subsequent crop was reduced and substitution of winter wheat with crops that received smaller amounts of fertilizer. In CB, the *optimized* system with and without legumes reduced nitrate leaching by 72–81% compared to common practice, because higher leaching was calculated for winter oat than for winter oilseed rape (Table 3). In BB and CB, a proportion of legume-supported systems resulted in negative gross margins because they did not include a sufficient proportion of profitable crops such as oilseed rape (Figure 2).

### Effects of integrating legumes into forage cropping systems

**Figure 3:**
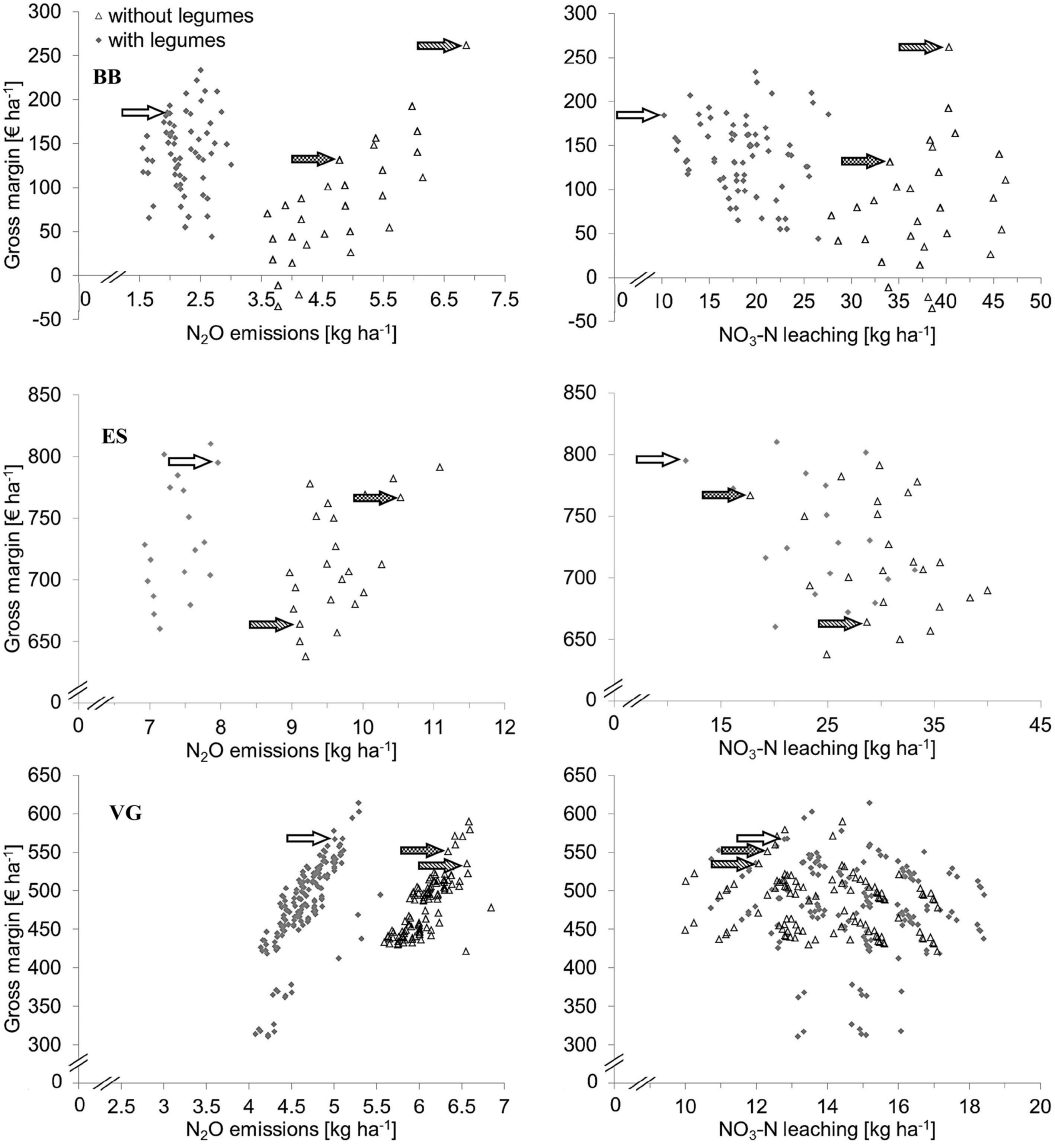
**Gross margin plotted against N_2_O emissions and NO_3_-N leaching for forage cropping systems with and without legumes in Brandenburg (BB), Eastern Scotland (ES), and Västra Götaland (VG)**. Blank and dotted arrows indicate economic-environmental optimized systems with and without legumes and dashed arrows indicate current farming.

The inclusion of forage legumes such as grass-clover and alfalfa and grain legumes in forage cropping systems reduced N fertilizer use by 27–58% and nitrous oxide emission by 23–52% (Table 2). The greatest reduction in N fertilizer use and nitrous oxide loss was found in BB where highly fertilized silage maize was replaced by unfertilized grass-clover. The effect was smaller in VG and ES where perennial grasses with low legume content were replaced by grass-clover including 30% clover in the crop biomass. Gross margins were similar between systems with and without legumes in VG and ES and 62% higher with legumes in BB (Table 2). Nitrate leaching was similar in both systems in VG and was reduced by the introduction of legumes in ES and BB. Similar to arable systems, forage cropping systems with high gross margins tended to have high nitrous oxide emissions, but no relationship was observed between gross margin and nitrate leaching (Figure 3).

The current cropping systems without legumes achieved high economic returns in BB and VG and resulted in relatively high nitrate leaching and nitrous oxide emission in all regions (Figure 3). In ES the *current farming* system with an additional cereal crop in the cropping system did not perform as well as the *optimized* systems economically or environmentally (Table 3), although it is practiced by a large proportion of farmers. Silage maize dominates *current farming* in BB, and has the best economic performance. Maize yields relatively well on these soils and the economic value of silage maize for feed and biogas is high. The *optimized* system without legumes performed better economically than *current farming* in ES and VG and better environmentally in all regions (Table 3). In BB, the *optimized* system with a lower share of silage maize resulted in improved environmental performance than the *current* system, but with lower gross margin.

The *optimized* system with legumes achieved 3–4% higher gross margins than without legumes in VG and ES because of the similar yield and price of grass-clover compared to grass, lower costs of fertilization, and a better pre-crop effect that led to a yield increase in the subsequent crop (Table 3). In BB, the integration of grass-clover resulted in 40% higher gross margin due to the high price of grass-clover forage, lower fertilizer costs and a greater pre-crop effect compared to silage maize, and despite the higher costs of harvesting 3 cuts of grass-clover rather than one crop of silage maize (Table 3). Across the regions, the *optimized* system with legumes had 23–54% lower N fertilizer use, 21–58% lower nitrous-oxide emissions, and, in BB, 70% lower nitrate leaching than that without legumes (Table 3). The major reasons for this difference include that no N was applied to the legume crop, the reduced N fertilizer doses to the subsequent crop and changes in the crop sequence.

### Trade-offs between economic and environmental impacts

**Figure 4:**
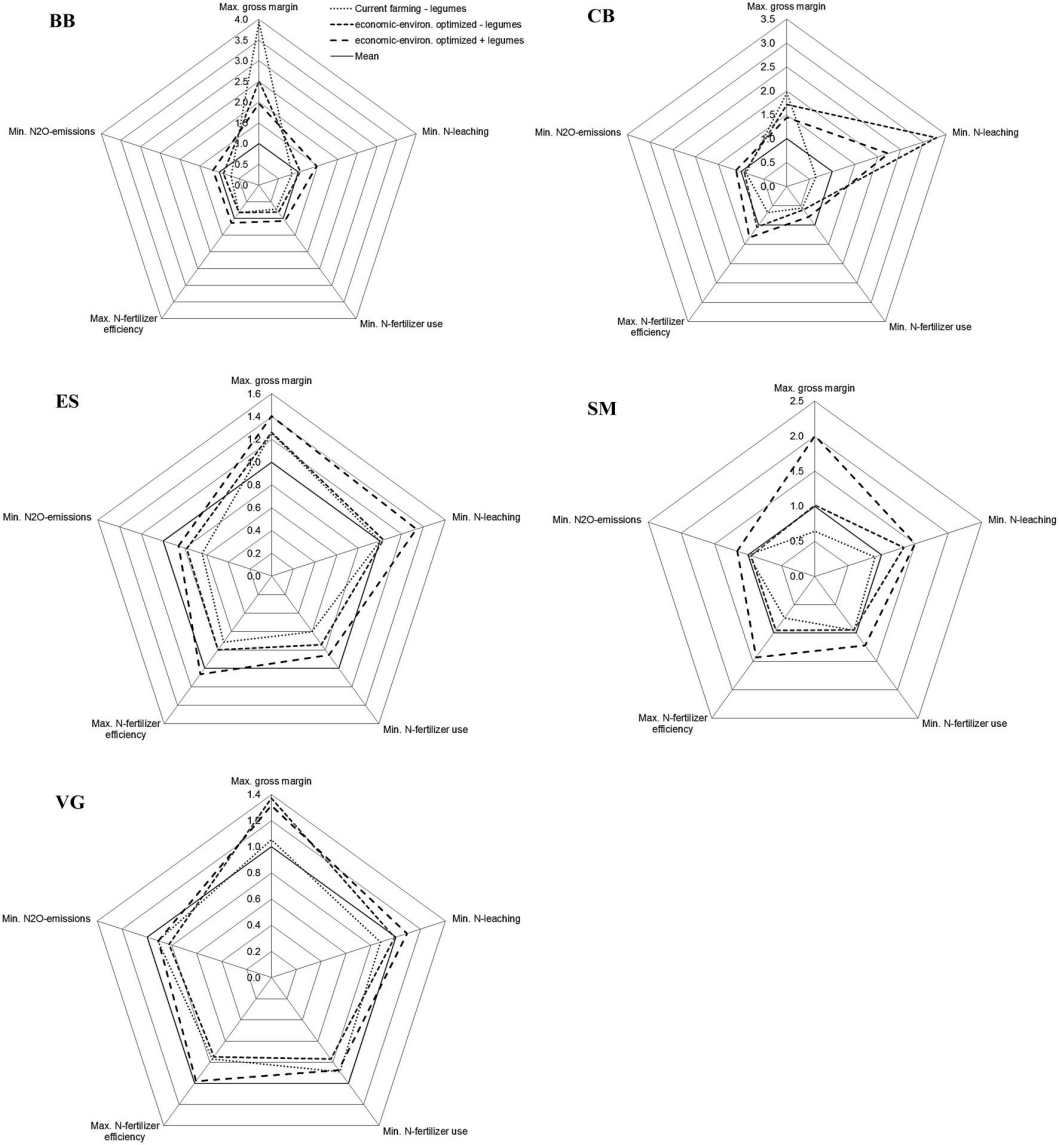
**Multi-criteria assessment of arable cropping systems in Brandenburg (BB), Calabria (CB), Eastern Scotland (ES), Sud-Muntenia (SM), and Västra Götaland (VG), for scenarios with and without legumes**. Values are the ratio of the single impact relative to the average impact calculated for that indicator across all cropping systems per region (outside values represent positive impacts). Absolute values are shown in Figure 2.

In all arable cropping systems, trade-offs were found between gross margin and environmental impacts (Figure 4). In BB, CB, and ES *current farming* had a high, above-average, economic and a low, below-average, environmental performance. The *optimized* system without legumes performed similarly in environmental terms to *current farming* in BB and ES. In CB, SM, and VG the *optimized* systems without legumes performed better on at least one of the environmental measures, particularly nitrate leaching. The *optimized* systems with legumes performed economically well in all regions, except in BB, and reached above-average scores for all environmental indicators (Figure 4).

**Figure 5:**
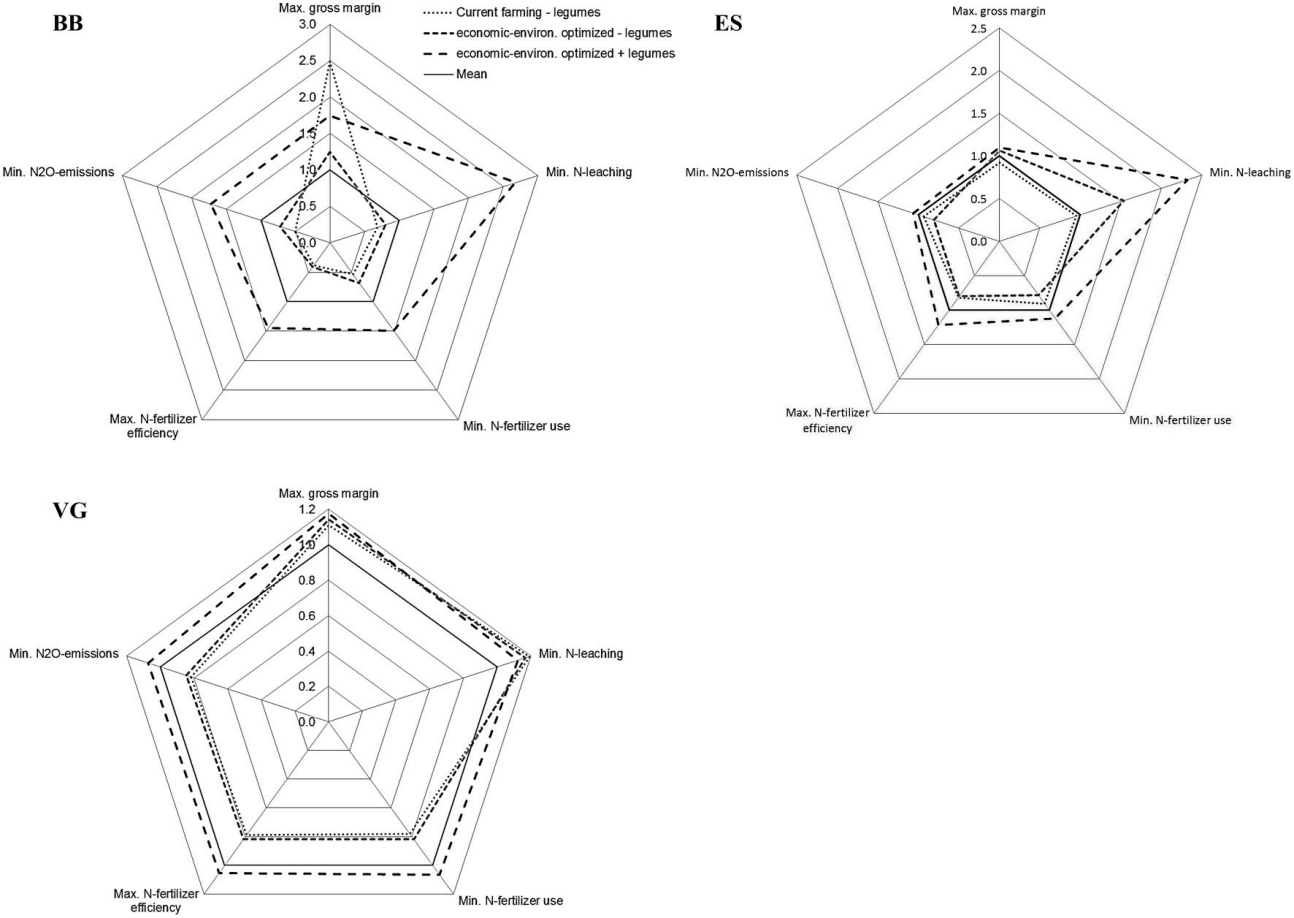
**Multi-criteria assessment of forage cropping systems in Brandenburg (BB), Eastern Scotland (ES), and Västra Götaland (VG), for scenarios with and without legumes**. Values are the ratio of the single impact relative to the average impact calculated for that indicator across all cropping systems per region (outside values represent positive impacts). Absolute values are shown in Figure 3.

Trade-offs between gross margin and environmental impacts were found for forage cropping systems without legumes (Figure 5). All systems without legumes performed below average, except regarding gross margin and nitrate leaching. The legume-supported systems reached above-average scores on all indicators, including gross margin. The environmental performance was generally good, especially considering nitrate leaching (Figure 5).

## Discussion

### Environmental and economic impacts

The reduction in the use of N fertilizer when legumes were integrated into cropping systems, 17–40% in arable and 27–58% in forage systems (Table 2), is mainly attributable to the nitrogen added through the BNF of legume crops (Peoples et al., 2009). The greatest savings were made in forage systems, because the perennial legumes included (clover and alfalfa) could fix more than 350 kg ha^−1^ (Carlsson and Huss-Danell, 2003), which is much more than the corresponding figures for the annual legumes, pea and faba bean, that fix about 130 kg ha^−1^ (Peoples et al., 2009). In BB, the N fertilizer savings were particularly large (58%), because unfertilized grass-clover replaced silage maize that received high doses of N fertilizer. In VG and ES, less N fertilizer was saved (27 and 29%, respectively) (Table 2), because the grass-clover was fertilized, even if the doses were quite low. The different management of grass-clover represents common farming practice in the respective regions. The potential of BNF in grass-clover could be exploited more effectively than is common practice in VG and ES if N fertilizer doses to grass-clover were reduced and/or distributed in time in a way that allows a sufficient share of legumes, as well as reducing losses (Eriksen et al., 2015). Nitrogen fertilizer savings in subsequent crops depend on the economic trade-off between securing maximum yields and maximizing N savings (Preissel et al., 2015). In our study, we used N fertilizer prices that were relatively low in relation to product prices. Therefore maintaining high yields came out as more important than saving N fertilizer, which means that we estimated small fertilizer savings, but high yield increases in subsequent crops. However, the framework allows new applications, such as to maximize N fertilizer savings, and the results of this analysis would have been different with this aim.

The average nitrous oxide emissions in cropping systems with legumes were 18 and 30% lower in arable and forage systems, respectively (Table 2). The differences among regions were greater for forage systems than for arable systems. The difference was greatest in BB (52%) due to the lower amounts of N applied to grass-clover than to silage maize. There is a direct relationship between N fertilizer input and nitrous oxide emissions, as shown by several studies compiled by Buckingham et al. (2014). However, we have used the assumption that 1% of each kg of N fertilizer is released as field-based nitrous oxide emission (IPCC, 2006), and this is currently under consideration. Philibert et al. (2012) calculated lower emission factors (EF) when the amount of N applied was below 160 kg N ha^−1^, Hinton et al. (2015) estimated EF to be between 0.28 and 1.35% of applied N depending on the N input, and Rees et al. (2012) concluded from a meta-analysis using measured data from Europe that annual emissions from arable sites were significantly greater than predicted by IPCC. Besides N fertilizer inputs, field-based nitrous oxide emissions are influenced by soil, management and environmental conditions (Ball et al., 2014). It seems clear that the EF used in the present study is approximate, but there is no consensus on how to modify it to better represent actual emissions.

The risk of nitrous oxide emission is generally considered to be lower from legumes than from cereals, oilseed rape and grassland (Stehfest and Bouwman, 2006; Schwenke et al., 2015). In a meta-analysis, Jensen et al. (2011) calculated that emissions from legume crops were around 40% lower than those from N-fertilized crops, and similar to unplanted soils or crops that were not N fertilized. The reason is that legume N inputs through BNF supplement the uptake of soil mineral N to meet crop N demand. The preferential use of soil mineral N leads to low availability of nitrate N for potential denitrification losses (Schwenke et al., 2015).

Nitrate leaching was similar with and without legumes in arable cropping systems (Table 2). Similarly to nitrous oxide emissions, nitrate leaching is soil-dependent and influenced by tillage, which did not differ between cropping systems with and without legumes. Tillage operations could in some circumstances be reduced before and after grain legumes (Luetke-Entrup et al., 2003; López-Bellido et al., 2003, 2004), which could reduce losses of nitrogen and increase gross margins of legume-supported systems (Preissel et al., 2015). The lower leaching in CB in the *optimized* cropping systems than in the current system was a result of the substitution of winter oat by winter oilseed rape (Table 3). The latter can take up more N before winter and thus reduce the risk of nitrate leaching (Sieling and Kage, 2012).

In forage systems, management factors such as soil tillage, nitrogen fertilization and the management of legumes in grass-clover mixtures influence nitrate leaching significantly (Eriksen et al., 2015). In our study, nitrate leaching was similar in both *optimized* systems in VG, slightly reduced with legumes in ES and strongly reduced in BB (Table 3). Reduced leaching with legumes in BB can be attributed to continuous crop cover in grass-clover compared to winter fallow between maize crops and high N fertilization of the maize crop compared to no N fertilization of grass-clover, in combination with a humid winter climate that causes most of the leaching to occur from October to March. In ES, lower doses of N fertilizer to grass-clover than to pure grass explain the slightly lower leaching.

The so-called break-crop effect is another aspect that increases the efficiency of utilizing N after a legume. Roots of a given crop are healthier after an unrelated crop has been grown, because pathogen populations are reduced (Angus et al., 2015), allowing more N to be taken up by the crop, and reducing the availability of N for leaching. There is a risk of large leaching losses after incorporation of N-rich crop residues, such as legume residues, so proper management is required to avoid losses (Eriksen et al., 2015). In our study, the risk of losses was reduced through the generation of optimized cropping systems where pre-crop effects were used effectively. Cover cropping is another option to reduce nitrate leaching after grain legumes (Plaza-Bonilla et al., 2015), but was not tested in this study.

Economic performance is regarded a key driver responsible for low adoption of legumes in cropping systems by farmers (Von Richthofen et al., 2006). As individual crops, legumes generally have lower gross margins than cereals and oilseed crops (Preissel et al., 2015). Our study revealed that the economic performance of legume crops was improved when assessed at the cropping system scale that allows consideration of break-crop effects on fertilizer use, other inputs, and yields of the following crops. The rotational effects of legumes are generally not considered in the economic evaluations of cropping systems (Zander et al., 2016). When cropping system effects are included, the competitiveness of legumes improved and the difference in gross margin between systems with and without legumes ranged from −67 to 106€ in arable and from 0 to 50€ in forage systems across all regions (Figures 2, 3). The high economic performance of legume systems in SM depends on stable market prices for common bean.

### Trade-offs between economic and environmental impacts

Cropping systems with a high economic performance tended to have high nitrous oxide emissions due to high input use. Such systems could be relevant for the intensification of agricultural systems, and concentrate the negative environmental impacts onto less arable land according to the land-sparing paradigm (Lamb et al., 2016).

The integration of grain legumes in arable systems had environmental benefits in all regions, but cropping systems with legumes also performed well economically in VG, SM, and ES (Figure 4). Thus the introduction of legumes in these regions added services without associated economic cost or losses in productivity, so they contributed to the ecological intensification of cropping systems (Doré et al., 2011). In CB and BB, the gross margin was lower in legume-supported systems than in those without legumes because of the low yields and prices of grain legumes.

Forage systems with legumes achieved both economic and environmental benefits in all regions because of relatively high yields, similar prices and better residual effects on subsequent crops considering yield and N fertilizer use (Figure 5).

Thus, adding a legume to cropping systems reduced trade-offs compared to systems without legumes resulting either in (a) win-win situations where legume cultivation is economically attractive and increases environmental benefits, or (b) situations where legumes are not economically attractive but increase environmental benefits.

### Opportunities and constraints for integrating legumes into cropping systems

In the case of win-win situations, legumes are already economically attractive and provide environmental benefits. The opportunities for forage legumes are easier to utilize because of (i) their generally better economic and environmental performance compared to annual grain legumes, (ii) their high quality feed value for livestock (relatively high prices), and (iii) their relatively simple integration into existing temporary grassland. The perennial nature of forage legumes offers opportunities for climate change mitigation and adaptation (Lüscher et al., 2014), improved biodiversity (Stein-Bachinger and Fuchs, 2012) and soil organic carbon content (Jensen et al., 2011), and lowered risk of both soil erosion (Jensen et al., 2011) and weed infestation (Håkansson, 2003). The deep rooting of some species improves subsoil accessibility for subsequent crops and increases the water infiltration rate (Fischer et al., 2014). However, the adoption of forage legumes is restricted to mixed farms with crop and livestock production, farm collaborations with livestock farmers, and farms that deliver green biomass to biogas plants (Tidåker et al., 2014). In mixed farms, nutrient management through the integration of crops and livestock remains a challenge, because of the high risk of nitrogen losses in manure management (Watson et al., 2005). Important agronomic constraints in legume production include crop establishment and to maintain about one third of legumes in grass-clover mixtures to achieve the maximum benefits from the clover (Suter et al., 2015). When feeding clovers and alfalfa to livestock, there is also an increased risk that ruminants suffer problems with digestion, i.e., bloat (Majak et al., 1995; Dewhurst et al., 2009). The specialization of farming and associated spatial decoupling of livestock and crop production is the major reason for the low proportion of forage legumes in Europe (Lemaire et al., 2015).

There are situations in arable systems were the introduction of legumes has both economic and environmental advantages, especially were grain legumes achieve high prices as a human food product (SM and ES) and where grain yields are relatively high. However, in most situations, grain legumes are still not competitive and there are multiple reasons why European farmers do not grow these crops. The major drivers are economic forces favoring cost-effective production systems and gains from international trade over the economic benefits of diversified production systems (Zander et al., 2016). Agronomic risks include lower yields and yield stability of grain legumes compared to cereals (Cernay et al., 2015), especially under conditions with low water availability such as in BB (Reckling et al., 2015) and in CB. The magnitude and causes of yield variability requires further research (Döring, 2015) and is attributable partly to the much lower investment in legume breeding than in cereal breeding (Meynard et al., 2013). Management strategies to reduce yield stability beyond good farming practices are limited. Other limitations mentioned by experts from the case study regions were increased weed infestation, root rot caused by *Aphanomyces euteiches* in pea, susceptibility to soil compaction, and lack of adapted cultivars (Stoddard, 2013). These constraints require that the legume crop be carefully selected to fit the local conditions and that the crop rotation be well designed with sufficient breaks between legumes that are affected by similar pathogens.

Marketing constraints for grain legumes are mainly due to low prices and few marketing channels. Prices are currently often lower than the actual feed value, and EU-produced grain legumes have difficulties competing with other protein crops on the world market (Voisin et al., 2013). The value chains for EU-produced grain legumes for feed and food are poorly developed in many European countries (Meynard et al., 2013). Regional supply chains for the feed and food market could increase competitiveness of legumes in Europe (Voisin et al., 2013) e.g., a GMO-free protein supply. Furthermore, ecosystem services, including reduced nitrous oxide emissions and nitrate leaching, and increased biodiversity are currently not rewarded through payments, so they are not considered in farmers' economic calculations and could justify policy support (Zander et al., 2016).

Farmers and advisors seldom consider the long-term benefits, focusing instead on single years. This leads to an underestimation of the services provided by legumes. The valuation of such services requires an assessment at the cropping-system scale (Reckling et al., 2016). Appreciation of these services could lead to wider adoption of legumes by farmers, but would also require that practical information on their rotational effects and proper management is available (Stoddard, 2013).

### Framework evaluation

The framework was evaluated using (i) design validation, (ii) plausibility checking of outputs and (iii) end-user validation, as proposed by Bockstaller and Girardin (2003) and described in detail by Reckling et al. (2016). The plausibility checking, especially through experts, provided sufficient information to adapt the model for the specific application in each of the contrasting case studies. Several iterations were needed with agronomists to define the input data and to evaluate the outputs. Thus the quality of the model outputs was dependent to a large extent on the quality of the expert knowledge, as is usually the case for rule-based models (Bachinger and Zander, 2007; Naudin et al., 2015).

The major sensitivity of the framework was the quantification of pre-crop effects that were estimated by experts and supported by information from field experiments (Reckling et al., 2016). Data from field experiments and dynamic models alone do not provide all the required data. Field experiments show varying effects between years and sites, and dynamic models have difficulties in handling pre-crop effects (Lorenz et al., 2013; Kollas et al., 2015).

The static calculation of nitrate leaching and nitrous oxide emission quantifies effects that do not consider the variability of weather, yields and environmental impacts (as is the case in process-based models), and are used here to make only relative comparisons between cropping systems. Nevertheless, static approaches are often used for nitrous oxide emission (Lokupitiya and Paustian, 2006; Berdanier and Conant, 2012) and nitrate leaching (Bachinger and Zander, 2007; Stein-Bachinger et al., 2015) when numerous systems are compared and measurements are not sufficiently available. The equation for nitrate leaching is based on the N surplus at the end of the growing season of each crop and might not sufficiently take into account all losses from mineralizing crop residues. This is especially the case when the N surplus is low due to high N uptake, e.g., the combination of high yields and low N input. Potential N losses through mineralization of N-rich residues from legumes after harvest until the end of the growing season might be underestimated. The indicators used for the evaluation were, however, found to be suitable to account for agronomic, environmental and economic aspects. Additional indicators such as erosion, soil organic matter, diseases and weed infestation, and the water foot print of crops could be added to the sustainability assessment in future.

The assessment framework generated and assessed large numbers of rotations that provide challenges to users, but the large number and diversity of systems provided the opportunity to explore cropping strategies outside existing system configurations and boundaries. This ability to explore a large number of options can also be used to complement information derived from life cycle assessments e.g., Nemecek et al. (2008). Through applying the selection criteria, we were able to identify novel *economic-environmental optimized* systems with legumes that could in the future be tested as prototypes by farmers (Vereijken, 1997).

A particular strength of this assessment framework is that it involves stakeholders from research and practical farming in the process of redesigning and assessing cropping systems that fulfill both economic and environmental aims, and that this can be done without having to test a wide range of systems on farms or experimental stations.

### Outlook

To utilize the multiple services of legumes, their production needs to increase significantly. Where legumes already lead to win-win situations with high economic and environmental benefits, such effects need to be communicated to advisors and farmers, and regional supply chains need to be developed for the feed and human food market.

In situations where legumes are not (yet) competitive but their environmental impact is considered desirable, constraints of low yield stability, weed and disease infestation, require innovative solutions from on-farm and on-station experimentation, use of crop management tools and advances in plant breeding. In such situations, policy support could be justified.

## Author contributions

All authors contributed to manuscript writing and read and approved the final manuscript. MR, GB, FS, CW, PZ, and JB designed the study. MR, PZ, and JB performed the modeling. RW, AP, IT, GB, MR, and JB provided data for the case studies and were involved in the validation of the results and interpretation.

## Funding

The work was financed by the German Federal Ministry of Food and Agriculture; the Brandenburg Ministry of Sciences, Research and Cultural Affairs; the Scottish Government RESAS Strategic Research Programme; the EU FP7 project “Legume Futures” (Grant 245216 CP-FP), and the FACCE-ERA-NET+ project Climate-CAFE (Grant PTJ-031A544). The publication of this article was funded by the Open Access fund of the Leibniz Association.

### Conflict of interest statement

The authors declare that the research was conducted in the absence of any commercial or financial relationships that could be construed as a potential conflict of interest.
